# Content and Communication of Inpatient Family Visitation Policies During the COVID-19 Pandemic: Sequential Mixed Methods Study

**DOI:** 10.2196/28897

**Published:** 2021-09-24

**Authors:** Joanna Hart, Amy Summer, Kuldeep N Yadav, Summer Peace, David Hong, Michael Konu, Justin T Clapp

**Affiliations:** 1 Division of Pulmonary, Allergy, and Critical Care Medicine Department of Medicine University of Pennsylvania Philadelphia, PA United States; 2 Leonard Davis Institute of Health Economics University of Pennsylvania Philadelphia, PA United States; 3 Palliative and Advanced Illness Research Center Department of Medicine University of Pennsylvania Philadelphia, PA United States; 4 Department of Medical Ethics and Health Policy University of Pennsylvania Philadelphia, PA United States; 5 Department of Anesthesia and Critical Care University of Pennsylvania Philadelphia, PA United States

**Keywords:** family-centered care, hospital policy, public health, health communication, ethics, health systems, public perceptions, COVID-19, pandemic

## Abstract

**Background:**

Inpatient health care facilities restricted inpatient visitation due to the COVID-19 pandemic. There is no existing evidence of how they communicated these policies to the public nor the impact of their communication choices on public perception.

**Objective:**

This study aims to describe patterns of inpatient visitation policies during the initial peak of the COVID-19 pandemic in the United States and the communication of these policies to the general public, as well as to identify communication strategies that maximize positive impressions of the facility despite visitation restrictions.

**Methods:**

We conducted a sequential, exploratory, mixed methods study including a qualitative analysis of COVID-19 era visitation policies published on Pennsylvania-based facility websites, as captured between April 30 and May 20, 2020 (ie, during the first peak of the COVID-19 pandemic in the United States). We also conducted a factorial survey-based experiment to test how key elements of hospitals’ visitation policy communication are associated with individuals’ willingness to seek care in October 2020. For analysis of the policies, we included all inpatient facilities in Pennsylvania. For the factorial experiment, US adults were drawn from internet research panels. The factorial survey-based experiment presented composite policies that varied in their justification for restricted visitation, the degree to which the facility expressed ownership of the policy, and the inclusion of family-centered care support plans. Our primary outcome was participants’ willingness to recommend the hypothetical facility using a 5-point Likert scale.

**Results:**

We identified 104 unique policies on inpatient visitation from 363 facilities’ websites. The mean Flesch-Kincaid Grade Level for the policies was 14.2. Most policies prohibited family presence (99/104, 95.2%). Facilities justified the restricted visitation policies on the basis of community protection (59/104, 56.7%), authorities’ guidance or regulations (34/104, 32.7%), or scientific rationale (23/104, 22.1%). A minority (38/104, 36.5%) addressed how restrictive visitation may impair family-centered care. Most of the policies analyzed used passive voice to communicate restrictions. A total of 1321 participants completed the web-based survey. Visitation policy elements significantly associated with willingness to recommend the facility included justifications based on community protection (OR 1.44, 95% CI 1.24-1.68) or scientific rationale (OR 1.30, 95% CI 1.12-1.51), rather than those based on a governing authority. The facility expressed a high degree of ownership over the decision (OR 1.16, 95% CI 1.04-1.29), rather than a low degree of ownership; and inclusion of family-centered care support plans (OR 2.80, 95% CI 2.51-3.12), rather than no such support.

**Conclusions:**

Health systems can immediately improve public receptiveness of restrictive visitation policies by emphasizing community protection, ownership over the facility’s policy, and promoting family-centered care.

## Introduction

During the global COVID-19 pandemic, health systems rapidly changed procedures and policies to minimize viral transmission and accommodate increased patient volumes and illness acuity [1]. One example of such a policy change is the restriction or elimination of family presence in inpatient settings [2-7]. The Centers for Disease Control and Prevention (CDC), the Center for Medicare and Medicaid Services, and national professional organizations recommended that health systems limit the presence of patients’ family members to reduce viral spread [2,8,9]. This recommendation reverses decades of policy and cultural evolution emphasizing the integral role of family members during inpatient care, based on research demonstrating that family presence during hospitalization improves patients’ and family members’ outcomes [2,3,10,11]. The language health systems use to communicate such policy changes likely shapes public perceptions of inpatient health care facilities at a time when there is an increased need for urgent medical care [12,13].

In the last two decades, the internet has become a critical vehicle in communicating information during public health emergencies [14]. During past crises, the public reported relying on health websites and physicians as trustworthy information sources. Prior work reveals that when the public lacks trust in a health care system, individuals are less willing to cooperate with health recommendations and seek the health services offered [15]. Communication during crises shapes individuals’ trust in health care systems through modifying their perceptions of the institutions’ competency, responsibility, and ability to fulfill its obligations to the public [16]. Therefore, effective communication of novel, crisis-era policies by health systems is critical to promoting public health and trust.

Crisis communication during a public health emergency is most useful when leveraged to improve community health and outcomes, rather than aimed to promote the reputation or image of an organization [16-18]. For example, the Crisis and Emergency Risk Communication (CERC) framework [19], which was developed by the CDC, merges both risk communication (ie, stakeholder-tailored communication focused on promoting behavior change) and crisis communication (ie, communication to reduce the negative effects of a crisis on stakeholders). CERC emphasizes that communication is critical to reducing health and psychological stressors during a crisis, in part through reestablishing a sense of personal control and encouraging self-efficacy. In this way, the current understanding of crisis communication is that its ultimate goal is not to inform the public but rather to develop high-quality social relationships [14,16,20].

Inpatient visitation policies are an example of crisis communication provided by health systems during the COVID-19 pandemic, and these frequently appear on health systems’ public websites. Despite widespread attention in the lay press [21-24], there is no published evidence systematically summarizing how health systems implemented national recommendations for restricted inpatient visitation, how they communicated these policies to the general public, and the impact of their communication choices on the public’s willingness to engage with the health care system. Therefore, we sought to characterize external communication made by inpatient health care facilities regarding their visitation policies during the COVID-19 pandemic. We aimed to identify key strategies for communicating such policies to improve their public reception [10]. To do so, we aimed to (1) describe the visitation policies of inpatient health care facilities during the COVID-19 pandemic, (2) classify patterns of how facilities communicated these policies to the public, and (3) identify associations between these communication patterns and public perception of the facility.

## Methods

### Overview

We conducted a sequential exploratory mixed-methods study [25]: first, we conducted a qualitative content analysis of visitation policies published on the internet by inpatient health care facilities during the COVID-19 era, followed by a factorial survey-based experiment to identify associations between elements of visitation policy communication and individuals’ perceptions of the facilities.

### Qualitative Analysis of Visitation Policy Communication

We identified inpatient health care facilities using the Pennsylvania Department of Health directory. We collected text and screenshots of each facility’s inpatient visitation policy from their websites between April 30 and May 20, 2020, during the first peak of COVID-19 cases in the United States and Pennsylvania (Multimedia Appendix 1) [26]. We eliminated duplicates of identical policies published by facilities within the same health system. We used Rural-Urban Commuting Area (RUCA) Codes to classify each facility as urban or rural (ie, RUCA >2) [27,28]. We assessed the readability of each policy using the Flesch-Kincaid Grade Level calculator [29]. This readability tool calculates an overall complexity of text using the mean sentence and word length, providing an overall score of educational school-grade levels 1 to 12 [30]. Scores >12 indicate a college-level education would be required to read the text.

Five investigators conducted qualitative analysis of the visitation policy statements. We first used qualitative content analysis, which sorts open-ended data using categories derived from close reading of texts; it is not associated with any particular theory or paradigm, but it is a generic means of categorizing the “informational content” of data [31]. We annotated the policy statements to identify the various functions they accomplished (eg, stating restrictions, justifying restrictions, expressing a facility’s values; Table 1). Within each of these function categories, we derived more specific categories to describe their content (eg, what the specific restrictions were, what information a facility presented to justify them, what specific values a facility expressed). Second, we performed discourse analysis—a method that examines the contextual use of language [32,33], to allow us to generate hypotheses about the effects of the specific language used to convey policies on readers. Through annotation, we observed that policies varied in how they positioned facilities as agentive or nonagentive in enacting restrictions through use of voice (active vs passive), sentence structure, and modality. Annotation was done independently and then discussed as a team. Based on this discussion, a formal codebook was generated to capture patterns in function, content, and discourse. Each policy was then coded using NVivo 12 qualitative analysis software (QSR International) by at least 2 team members (AS, SP, DH, and MK), with discrepancies resolved and codebook refinements made by consensus and consultation with supervising investigators (JH and JC). Finally, we performed focused coding, prioritizing the codes that we believed were most important to how visitation policies were communicated, identifying relationships between these codes, and synthesizing related codes [34-38]. Focused coding was done through group discussion and captured via analytic memos.

### Factorial Experiment of Visitation Policy Communication

We used the results of the qualitative analysis to develop a factorial survey-based experiment that tested the impact of variation in hospitals’ public visitation policy communication. We selected three elements to vary based on our qualitative findings by generating a list of elements that varied and eliminating those well-established to influence public opinion and understanding (eg, reading level) or those that health systems may be unable or unwilling to change (eg, the visitation rules themselves). Our final selections included (1) how the inpatient facility justified restricting visitation (eg, community protection, regulations from a governing authority, or scientific rationale); (2) the degree to which the facility expressed ownership over the policy decisions (eg, use of active voice to indicate responsibility for decision-making); and (3) the inclusion or absence of family-centered care support plans (eg, virtual visits or team communication plans and expressions of concern for well-being). Although actual policies justifying the restricted visitation on the basis of governing authorities more frequently used passive voice, in our factorial experiment, these varied independently.

Our final 3×2×2 factorial experiment structure generated 12 hypothetical policies (Multimedia Appendix 1). For our visitation policy statements presented to participants in the survey, we used the most common visitation rules and language drawn from actual facility policies in a composite manner to represent our selected elements. We used a Flesch-Kincaid Grade Level range of 9.0 to 10.8 for all policies. This was lower than the average policy in our sample, but it remains above the recommended reading level for patient education materials, as we used facilities’ actual word choices whenever possible for constructing the composite policies [39,40]. We reviewed our element selections and the survey with groups of clinical and nonclinical research staff and pilot tested the survey with nonclinical individuals prior to launch.

We recruited participants in October 2020, during the start of the second and largest peak of COVID-19 cases in the United States (Multimedia Appendix 1). We used Cloud Research’s Prime Panels, an internet study recruitment platform that enables and ensures high-quality survey-based data collection from the over 50 million diverse enrolled participants who more closely represent the US population than other similar platforms, such as MTurk [41]. The platform also allows for recruitment based on targeted eligibility criteria. Potential participants were eligible to voluntarily choose to complete this study if they resided in the United States, were ≥18 years old, and were fluent in written English. We conducted stratified recruitment to ensure that a minimum of 44% of participants were women and 13% were Black or African American to reflect US demographics more closely [41]. We asked all participants to complete a survey that included reviewing the 12 hypothetical policies and responding after each using a 5-point Likert scale (ie, ranging from “1=not at all” to “5=very much”), indicating the participant’s willingness to recommend the hospital on this basis alone. They then responded to survey items collecting their personal perspectives on COVID-19 risk and mitigation strategies, the 9-item Revised Healthcare System Distrust Scale (HSDS) [42], and a demographic questionnaire. We included three attention checks and participants who failed two of these checks were no longer eligible to be included in the analysis [41,43]. Participants received a nominal compensation for successful survey completion. All participants provided informed consent after reviewing written information prior to beginning the survey. All study procedures were approved by the University of Pennsylvania’s Institutional Review Board.

Using the n.multiway function from the R package easypower, we calculated sample size estimates of our 3×2×2 factorial design based on the number of factors (ie, policy elements), the number of levels of each factor, and various effect sizes for a main effect of a factor or for the interaction between two factors on the willingness to recommend the hospital (Table S1 in Multimedia Appendix 1). We estimated that recruiting 1272 participants (ie, 106 for each of 12 hypothetical policies) would be sufficient to detect an odds ratio (OR) of 1.437 for the main effect or interaction with 90% power and an α=.05.

We examined the relationship among the three policy elements and willingness to recommend the hospital using mixed effects logistic regression. The participant was entered as a random effect to account for the clustering of responses by each participant [44]. The regression model was fit using maximum likelihood estimation and was implemented using the glmer function from the R package lme4 [45]. Prior to model building, we determined that continuous variables were appropriate to enter as linear terms without transformation. The unadjusted associations between all predictor variables and the primary outcome were examined using *t* tests and chi-square tests for continuous and categorical variables, respectively. A priori, we decided to include all three tested elements in the baseline model. Other variables were selected for inclusion using backward variable selection in a stepwise manner from a model with all possible covariates. Those that were not significant (eg, age and gender of the participant) were not included in the final model given we had no prespecified hypotheses as to the direction of effect. The final model only included predictor variables with *P* values <.05 for the association between the variable and the primary outcome in the baseline model, including the three exposure variables [46]. We explored potential interactions among all three visitation policy elements by adding two-way interaction terms one at a time to the final model, but none were significant. Two-sided *P* values <.05 were considered statistically significant. All statistical analyses were conducted in R Studio.

## Results

### Qualitative Analysis of Visitation Policy Communication

We identified 513 inpatient health care facilities within Pennsylvania. Using RUCA classification, 80.3% (n=412) were urban and 20.7% (n=101) were rural. We identified inpatient visitation policies for 363 facilities, resulting in 104 unique policies after eliminating duplicates used for multiple facilities within a health system (Figure S1 in Multimedia Appendix 1). The mean Flesch-Kincaid Grade Level for the policies was 14.2 (ie, requiring college-level reading skills). In addition to English, the policy was available in Spanish for 2 of the 104 (1.9%) unique policies, representing 1.1% (4/363) of all facilities’ policies.

Nearly all policies restricted inpatient visitation. A minority (29/104, 27.9%) prohibited all visitors, whereas most (70/104, 67.3%) identified exceptions permitting some visitation. Few (5/104, 4.8%) policies specified no restrictions. Of the 70 policies allowing exceptions, 37 (52.9%) offered standardized exceptions (eg, end-of-life care, childbirth, and pediatric patients), 16 (22.9%) offered case-by-case exceptions, 13 (18.6%) included both, and 4 (5.7%) included no information about exceptions. Other common rules were restrictions on visitors’ movement through the facility and mandatory screening (eg, symptom checks) prior to entry.

There were 8 major functions of phrases within the published policies (Table 1), including communication of rules and exceptions, justification of the restrictive policy, indication that the policy was a deviation from normal procedures, statements of the facilities’ values, and a recognition of the hardship that these policies cause for patients and their families. Most (85/104, 81.7%) policies included a justification, most commonly the protection of patients, staff, or community (59/104, 56.7%); guidance or regulations from governing or scientific authorities (34/104, 32.7%); or a scientific rationale (23/104, 22.1%). A minority (38/104, 36.5%) included a statement recognizing that these policies may distress families and patients or that the delivery of family-centered care may be impaired. Governmental agencies at the state or federal level (eg, the Centers for Disease Control and Prevention or the Pennsylvania Department of Health) were named as responsible for the restrictive policies. Policies rarely included an explanation of how restricting visitation relates to viral transmission.

Most policy statements delineating the visitation rules or indicating that the restrictions were a deviation from typical procedures used the passive voice (Table 2). Statements addressing resulting hardship, stating the facilities’ values, or emphasizing the temporary nature of the policy used the active voice. Policies that justified restricted visitation on the basis of protection generally used the active voice, whereas those that justified restrictions due to authorities used passive voice.

**Table 1:** Function of statements within policies.

Function of statement	Example
Indicate that the policy is temporary	“[FACILITY] has put into place a temporary hospital and outpatient visitation policy”
Acknowledge that the policy differs from standard hospital policy	“This policy replaces our traditional open visitation policy”
State the rules themselves	“In end-of-life situations [FACILITY] will allow loved ones to visit with patients”
Justify implementation of the policy	“We are now increasing these restrictions due to federal and state recommendations related to COVID-19”
Recognize the hardship that the policy brings for patients and families	“We know that the no visitor policy may be upsetting for our patients, residents, and their families”
Emphasize the values driving the facility or health system	“The health and well-being of the children and adults we serve remains our top priority in every decision we make”
Identify where patients and potential visitors can obtain additional information	“Please call the [FACILITY] COVID-19 Information Line (XXX-XXX-XXXX) for clarification and additional details”

**Table 2:** Degree of ownership displayed within and across policy statements.

Statements demonstrating high or low ownership over policy decisions and implementation		Examples
**High ownership**		
	Indicate that the policy is temporary	“We will regularly re-evaluate these visitor restrictions as we receive updates about COVID-19”
	Recognize hardship that policy brings for patients and families	“We understand the importance of the support of friends and family to the healing process”
	Emphasize values driving the facility or health system	“[FACILITY] is committed to the health, safety, and well-being of the communities we serve”
	Offer alternatives to in-person family presence	“We strongly encourage the use of electronic methods to stay connected with loved ones including telemedicine, zoom, and extended phone time”
	Justify policy implementation based on protection and safety	“Be assured that we are making these decisions in your best interest so that we can ensure the safety of you, your baby, and our staff”
**Low ownership**		
	Acknowledge that the policy differs from standard hospital policy	“Beginning Tuesday, March 24, inpatient and outpatient visitation guidelines will shift from limited to restricted, outlined below”
	State the policy itself	“All in-person visitation has been suspended (with limited critical exceptions)”
	Justify the policy based on authority guidance and regulations	“This restriction has been implemented in compliance with updated corporate and state regulations to further reduce the risks associated with COVID-19”

### Factorial Experiment of Visitation Policy Communication

In total, 6272 individuals in the Cloud Research Prime Panel system may have viewed the study description, and 1602 individuals may have opened the study link. Of these, 1356 participants completed the instrument for a conservatively estimated response rate of 25.5%. We excluded 34 responses due to failed attention checks. The 1321 participants in the final analysis had a median completion time of 9.2 minutes (IQR 6.4-14.2 minutes). Participants’ characteristics are summarized in Table 3. A minority of individuals self-reported high personal risk of COVID-19 or having close contact with an individual at high risk of COVID-19. The majority were insured and demonstrated moderate to low levels of health care system distrust [42].

**Figure 1:**
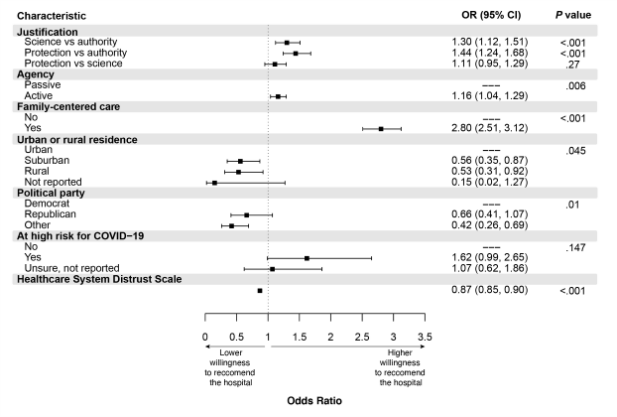
Association of policy elements and respondent characteristics with willingness to recommend the hospital. OR: odds ratio.

Results of the multivariable model are displayed in Figure 1 and Table S2 of Multimedia Appendix 1. Visitation policy elements significantly associated with a willingness to recommend the hospital included justifications for restricted visitation based on community protection (OR 1.44, 95% CI 1.24-1.68) or scientific rationale (OR 1.30, 95% CI 1.12-1.51), in contrast to regulations from a governing authority. The hospital displayed a high degree of expressed ownership over the policy (OR 1.16, 95% CI 1.04-1.29 vs low degree of ownership) and inclusion of family-centered care support plans (OR 2.80, 95% CI 2.51-3.12 vs absence of such support). Justification of visitation restrictions to support community protection was significantly more likely to result in willingness to recommend the hospital when compared with scientific rationale for restrictions in the pairwise comparisons from the final model (Figure 1). In the multivariable model, participants living in urban settings, reporting more trust in the health care system, and identifying as Democrats were significantly more willing to recommend the hypothetical hospitals after adjusting for visitation policy elements (Figure 1).

**Table 3:** Characteristics of survey participants.

Characteristic		Participants (N=1321)^a^
Age (years), median (IQR)		39 (30-55)
**Gender, n (%)**		
	Women	710 (53.7)
	Men	601 (45.5)
	Transgender, nonbinary, other	10 (0.8)
**Race, n (%)**		
	White, Caucasian American	967 (73.2)
	Black, African American	248 (18.8)
	Other^b^	106 (8)
**Ethnicity, n (%)**		
	Not Hispanic, Latinx	1195 (90.5)
	Hispanic, Latinx	115 (8.7)
	Not reported	11 (0.8)
**Educational attainment^c^, n (%)**		
	Less than high school, GED^d^	269 (20.4)
	Some college	383 (29)
	Bachelor's degree	385 (29.1)
	Graduate degree	284 (21.5)
**Urban or rural residence**, **n (%)**		
	Urban	535 (40.5)
	Suburban	514 (38.9)
	Rural	261 (19.8)
	Not reported	11 (0.8)
**Political party, n (%)**		
	Democrat	566 (42.8)
	Republican	396 (30)
	Other^e^	359 (27.2)
**Insurance status, n (%)**		
	Insured	1207 (91.4)
	Uninsured	114 (8.6)
**At high risk for COVID-19, n (%)**		
	No	775 (58.7)
	Yes	325 (24.6)
	Unsure, not reported	221 (16.7)
**Close contact with someone at high risk for COVID-19, n (%)**		
	No	856 (64.8)
	Yes	358 (27.1)
	Unsure, not reported	107 (8.1)
**Revised HSDS^f^ score**		
	Mean (SD)	22.4 (7.2)
	Median (IQR)	24 (17-28)

^a^Percentages may not add up to 100% due to rounding. For gender and HSDS (n=1320), 1 participant did not provide responses.

^b^For race, “Other” includes Asian, Asian American, Pacific Islander, Native American, Hispanic/Latino, Mestizo, Middle Eastern, Romanian, and multiracial.

^c^For education attainment, “Some college” includes associate's or professional certificate; “Graduate degree” includes master's, doctoral, or professional degree.

^d^GED: general educational development.

^e^For political party, “Other” includes independent, democratic socialist, libertarian, and no political affiliation.

^f^HSDS: Healthcare System Distrust Scale. For the revised HSDS (9-item scale; range: 5-45), higher total scores indicate more distrust of the health care system.

## Discussion

### Principal Findings

Although health care systems across the globe have restricted inpatient family visitation during the COVID-19 pandemic [3-5], there is a lack of empirical research presenting what visitation rules health systems enacted, how health systems communicated these policies to the lay public, and the impact of those communication choices on public opinion. In this study, we present a mixed methods investigation including qualitative analysis of publicly available COVID-19 era inpatient visitation policies and a subsequent factorial experiment examining the impact of three key policy statement elements on lay individuals’ willingness to recommend the hypothetical hospital to a family member. Most of the website-published policies prohibited family presence and justified these restricted visitations on the basis of community protection over authorities’ guidance, regulations, or scientific rationale. A few policies addressed how restrictive visitation may impair family-centered care.

Our findings confirm the importance of communication choices during periods of crisis [12,13]. Importantly, we identify specific elements of visitation policy statements strongly associated with individuals’ willingness to recommend the hospital. Because these are unrelated to the visitation rules themselves, health systems may rapidly implement these findings.

The goals of family-centered care are to respect family members as care partners, foster collaborations between family members and the health care team, and maintain family integrity [2,10]. There is broad recognition among clinicians and the general public that visitation policies restricting family presence for inpatients are undesirable and detrimental to family-centered care [2,3,5,21-24,47]. Our participants valued the inclusion of family-centered care support plans and recognition of resulting distress in hospitals’ communication of visitation restrictions. Indeed, family-centered content was more strongly associated with individuals’ willingness to recommend a hospital than any other element. However, few inpatient facilities’ policy statements provided resources to promote family members’ engagement with admitted patients or health care teams or expressed empathy and compassion. Including empathy in crisis messages is known to result in a more positive public response and enhances the credibility of the messenger [12]. Health systems should prioritize communication and coping resources for family members distanced from inpatients during and after pandemic conditions and clearly communicate the existence of these resources to engender trust among community members [2].

Inpatient facilities’ frequent use of the passive voice to communicate restrictive visitation rules suggests that they seek to distance themselves from unfavorable policies. For example, facilities frequently justified restrictive policies by referring to governing authorities while using the passive voice. When facilities used the active voice to express a high degree of ownership in the policies, they did so for favorable components of the policy statements. These patterns suggest that facilities may attempt to deflect responsibility for undesirable visitation restrictions. However, our findings indicate this is likely counterproductive. Individuals were more likely to recommend a facility that expressed ownership of restrictive policies, including providing a justification that did not rely on governing authorities and using the active voice when communicating restrictive rules. Community members may interpret facilities as being connected and invested in the communities they serve if systems express a high degree of ownership over decision-making, rely on community protection as a salient rationale for restrictions, and support family-centered care despite visitation restrictions.

Many of the inpatient facilities’ policy statements did not reflect best practices in community and crisis communication [12]. Many failed to engage in two-way risk communication, in which the public is treated as full participants rather than passive information receivers or rule followers [16,48,49]. Such two-way risk communication strategies focus on building dialogue with public citizens who have rights and values. This can lead to improved public understanding of the risks and rationale for policies and recommendations. Furthermore, this approach to communication positions the rule-makers as transparent and worthy of trust. In contrast, facilities rarely explained the mechanisms through which restrictive family presence policies may reduce the transmission of COVID-19. Particularly early in the pandemic, when we collected the policies, community members likely did not have this scientific knowledge, limiting their ability to participate in the dialogue around visitation restrictions [50]. Additionally, policy statements intending to convey key messages to the general public should be written at a reading level accessible to those with low reading levels, given that the average American reads at an 8th grade reading level [39,40,51]. We elected not to test varied reading levels in our factorial design given the large body of work that exists supporting the need for accessible public health information [51], particularly during the COVID-19 crisis as the burden of impact has been disproportionately high in medically underserved communities [40,52-54]. Policy statements that require a college reading level are likely to contribute to misunderstandings and lack of trust [39,40,51,55]. Health systems should also offer their materials in non-English languages that are spoken by community members, yet this was rarely observed in our sample.

Although the communication of these crisis-era policies should be improved upon, an organization’s pre-crisis credibility is critical to the public’s trust in the organization during a crisis [12]. If a health system or facility has not already established itself as supportive of families, is not engaged with community health, and is not concerned about staff well-being, the organization’s statements of these values may be meaningless. Therefore, the approach to communicating policies during COVID-19 and similar crises should also reflect a broader attention to the relationship between health systems and their surrounding communities both during and between acute public health crises.

### Limitations

Our findings should be interpreted in the context of several limitations. First, we gathered policy statements from a single state. However, we did seek to capture all publicly available policies to eliminate selection or response bias introduced with other methods, such as surveys. Second, we captured policy statements immediately following the initial peak of COVID-19 cases in Pennsylvania, so our analysis does not reflect longitudinal changes to the policy statements or visitation rules [26]. We cannot comment on longitudinal trends in visitation policies enacted by inpatient facilities nor public perception of these policies in the context of national or local COVID-19 burden or governmental policies intended to control viral spread. Third, we were unable to find policies for all licensed inpatient facilities in Pennsylvania. However, we used widely available search engines, mitigating the risk that the public had access to an internet-published policy we did not locate. Fourth, the public may not read or compare policies available on websites. Nevertheless, the majority of inpatient facilities had internet-published policies, suggesting they believe there is an audience for this information. Fifth, our factorial experiment relied on participants from an internet research panel, which may not reflect the general US population [36]. We took steps to mitigate the risk of bias, including stratified recruitment on the basis of race and gender [49]. Sixth, we selected elements to vary in the policies for the factorial experiment. There may be alternative elements more important than the ones we selected. However, we selected elements based on our preceding content analysis, making our findings valuable even if other elements are considered important. Seventh, we tested composite policy statements without contextual factors that may lead a community member to recommend or discourage a particular hospital, and we did not require participants to choose between hospitals based on the policy statements, which may have limited variation in their responses. Despite this, we identified several significant findings.

### Conclusions

Inpatient facilities frequently enacted restrictive inpatient visitation policies during the COVID-19 pandemic. Communication of these policies did not reflect best practices in crisis communication and may fail to represent health systems’ commitment to their communities’ health and their motives for establishing restrictive visitation policies. To improve public perception, health systems and facilities should convey ownership over their policy decisions, justify unfavorable visitation rules based on community protection, and include resources supporting family-centered care. Policies should also use language accessible to those with lower reading levels and in languages other than English as locally relevant, provide clear explanations for novel policies, and more frequently incorporate statements of empathy and compassion when communicating crisis-era policies affecting the general public.

## Appendix

Multimedia Appendix 1 Timeline of local COVID-19 burden during the study period; survey instrument used; sample size estimates; identification of unique inpatient facilities’ visitation policies; and multivariable model.

## Abbreviations

CERC: Crisis and Emergency Risk Communication
HSDS: Healthcare System Distrust Scale
RUCA: Rural-Urban Commuting Area
