# Supplementary material for: Content and Communication of Inpatient Family Visitation Policies During the COVID-19 Pandemic: Sequential Mixed Methods Study
Source: J Med Internet Res. 2021 Sep 24;23(9):e28897. doi: 10.2196/28897 (PMC8477908; doi:10.2196/28897)
Supplement: Multimedia Appendix 1 [file jmir_v23i9e28897_app1.docx]

Multimedia Appendix 1

- Timeline of local COVID-19 burden during study period
- Survey instrument
- Table S1: Sample size estimates
- Figure S1: Identification of unique inpatient facilities’ visitation policies
- Table S2: Multivariable model

**Timeline of local COVID-19 burden during study period**

**State of Pennsylvania (March to July 2020):**

March 6, 2020: first COVID-19 cases reported

March 13, 2020: all schools in the state closed by state mandate

March 16, 2020: state-wide social distancing mandates put into place

March 19, 2020: all non-essential businesses closed by state mandate

April 1, 2020: state-wide shelter-at-home mandate put into place

[April to May 2020: visitation policies collected for this study]

May 27, 2020: US deaths due to COVID-19 total over 100,000 to date

July 1, 2020: state-wide mask mandate put into place

**United States (September to December 2020):**

September 22, 2020: US deaths due to COVID-19 total over 200,000 to date

October 2, 2020: US President Trump develops COVID-19, requiring hospitalization

[October 2020: factorial experiment conducted among US residents]

November 8, 2020: US reports over 10 million people have been infected with SARS-CoV-2, with 1 million new cases in the prior 10 days

November 18, 2020: US deaths due to COVID-19 total over 250,000 to date

Source for US information: Derrick Bryson Taylor, A Timeline of the Coronavirus. New York Times, March 27, 2021.

**Survey Instrument**

During the COVID-19 pandemic, hospitals have put in place new policies and procedures. We would like to understand your reactions to sample statements communicating these new policies.

You will be asked to read 12 brief statements from different hospitals that might appear on their public websites. Please read each statement completely, as they are all different.  After reading, please respond to the questions following each statement.

*Policies were provided in randomized order to participants. Annotations (coloring) were not present in the policies seen by participants. Green passages indicate inclusion of family-centered care. Blue passages indicate justification of the restrictions. The first six policies are written in active voice and indicate a high degree of decision making by the facility. Examples of this are shown in yellow.*

A. We understand how much it means to you to be able to visit with your loved ones while they are in the hospital - and how much it means for them to have you by their side. Unfortunately, we are restricting in-person hospital visitation until further notice. We do not allow family members to visit hospitalized patients. We do allow one visitor per patient in special circumstances: patients under the age of 18, labor and delivery and postpartum patients, patients with disabilities or special needs, and patients at the end of life. We made the decision to limit visitors based on guidance and regulations issued by the local and state Departments of Health and the United States Centers for Disease Control and Prevention (CDC). We have ongoing review processes in place and will update our policies to follow any changes to their recommendations. We encourage you to use FaceTime, Skype, Zoom, and phone calls to virtually visit with loved ones while they are in the hospital. We have increased the supply of iPads and tablets for patients to use, and staff members will help them with these virtual visits.

B. Unfortunately, we are restricting in-person hospital visitation until further notice. We do not allow family members to visit hospitalized patients. We do allow one visitor per patient in special circumstances: patients under the age of 18, labor and delivery and postpartum patients, patients with disabilities or special needs, and patients at the end of life. We made this decision based on guidance and regulations issued by the local and state Departments of Health and the United States Centers for Disease Control and Prevention (CDC). We have ongoing review processes in place and will update our policies to follow any changes to their recommendations.

C. We understand how much it means to you to be able to visit with your loved ones while they are in the hospital - and how much it means for them to have you by their side. Unfortunately, we are restricting in-person hospital visitation until further notice. We do not allow family members to visit hospitalized patients. We do allow one visitor per patient in special circumstances: patients under the age of 18, labor and delivery and postpartum patients, patients with disabilities or special needs, and patients at the end of life. We made this decision as we are working hard to keep our patients, staff, and community safe during the pandemic. Our first priority is to protect the health of those we serve and we take that responsibility seriously. We encourage you to use FaceTime, Skype, Zoom, and phone calls to virtually visit with loved ones while they are in the hospital. We have increased the supply of iPads and tablets for patients to use, and staff members will help them with these virtual visits.

D. Unfortunately, we are restricting in-person hospital visitation until further notice. We do not allow family members to visit hospitalized patients. We do allow one visitor per patient in special circumstances: patients under the age of 18, labor and delivery and postpartum patients, patients with disabilities or special needs, and patients at the end of life. We made this decision as we are working hard to keep our patients, staff, and community safe during the pandemic. Our first priority is to protect the health of those we serve and we take that responsibility seriously.

E. We understand how much it means to you to be able to visit with your loved ones while they are in the hospital - and how much it means for them to have you by their side. Unfortunately, we are restricting in-person hospital visitation until further notice. We do not allow family members to visit hospitalized patients. We do allow one visitor per patient in special circumstances: patients under the age of 18, labor and delivery and postpartum patients, patients with disabilities or special needs, and patients at the end of life. Due to community spread of the virus in our area, visitors to patients in our hospitals have the potential to both carry and spread infection. Because many people infected with the COVID-19 virus have no symptoms and the virus is highly infectious by face-to-face contact, we must limit the number of people in our hospital. We encourage you to use FaceTime, Skype, Zoom, and phone calls to virtually visit with loved ones while they are in the hospital. We have increased the supply of iPads and tablets for patients to use, and staff members will help them with these virtual visits.

F. Unfortunately, we are restricting in-person hospital visitation until further notice. We do not allow family members to visit hospitalized patients. We do allow one visitor per patient in special circumstances: patients under the age of 18, labor and delivery and postpartum patients, patients with disabilities or special needs, and patients at the end of life. Due to community spread of the virus in our area, visitors to patients in our hospitals have the potential to both carry and spread infection. Because many people infected with the COVID-19 virus have no symptoms and the virus is highly infectious by face-to-face contact, we must limit the number of people in our hospital.

G. It is meaningful for family members to be able to visit with loved ones while they are in the hospital - and it is meaningful for patients to have family members by their side. Unfortunately, in-person hospital visitation is restricted until further notice. No visitors are allowed for most patients. One visitor per patient is allowed in special circumstances: patients under the age of 18, labor and delivery and postpartum patients, patients with disabilities or special needs, and patients at the end of life. This follows the guidance and regulations issued by the local and state Departments of Health and the United States Centers for Disease Control and Prevention (CDC). Family members may use FaceTime, Skype, Zoom, and phone calls to virtually visit with loved ones while they are in the hospital. There is an increased supply of iPads and tablets for patients to use and staff members will help them with these virtual visits.

H. Unfortunately, in-person hospital visitation is restricted until further notice. No visitors are allowed for most patients. One visitor per patient is allowed in special circumstances: patients under the age of 18, labor and delivery and postpartum patients, patients with disabilities or special needs, and patients at the end of life. This follows the guidance and regulations issued by the local and state Departments of Health and the United States Centers for Disease Control and Prevention (CDC).

I. It is meaningful for family members to be able to visit with loved ones while they are in the hospital - and it is meaningful for patients to have family members by their side. Unfortunately, in-person hospital visitation is restricted until further notice. No visitors are allowed for most patients. One visitor per patient is allowed in special circumstances: patients under the age of 18, labor and delivery and postpartum patients, patients with disabilities or special needs, and patients at the end of life. These limits are necessary to keep the patients, staff, and community safe during the pandemic. Limiting visitors protects the health of those who rely on the hospital. Family members may use FaceTime, Skype, Zoom, and phone calls to virtually visit with loved ones while they are in the hospital. There is an increased supply of iPads and tablets for patients to use and staff members will help them with these virtual visits.

J. Unfortunately, in-person hospital visitation is restricted until further notice. No visitors are allowed for most patients. One visitor per patient is allowed in special circumstances: patients under the age of 18, labor and delivery and postpartum patients, patients with disabilities or special needs, and patients at the end of life. These limits are necessary to keep the patients, staff, and community safe during the pandemic. Limiting visitors protects the health of those who rely on the hospital.

K. It is meaningful for family members to be able to visit with loved ones while they are in the hospital - and it is meaningful for patients to have family members by their side. Unfortunately, in-person hospital visitation is restricted until further notice. No visitors are allowed for most patients. One visitor per patient is allowed in special circumstances: patients under the age of 18, labor and delivery and postpartum patients, patients with disabilities or special needs, and patients at the end of life. Due to community spread of the virus in our area, visitors to hospitalized patients have the potential to both carry and spread infection. Because many people infected with the COVID-19 virus have no symptoms and the virus is highly infectious by face-to-face contact, there must be limits on the number of people in the hospital. Family members may use FaceTime, Skype, Zoom, and phone calls to virtually visit with loved ones while they are in the hospital. There is an increased supply of iPads and tablets for patients to use and staff members will help them with these virtual visits.

L. Unfortunately, in-person hospital visitation is restricted until further notice. No visitors are allowed for most patients. One visitor per patient is allowed in special circumstances: patients under the age of 18, labor and delivery and postpartum patients, patients with disabilities or special needs, and patients at the end of life. Due to community spread of the virus in our area, visitors to hospitalized patients have the potential to both carry and spread infection. Because many people infected with the COVID-19 virus have no symptoms and the virus is highly infectious by face-to-face contact, there must be limits on the number of people in the hospital.

|  | Not at all (1) | (2) | (3) | (4) | Very much (5) |
| --- | --- | --- | --- | --- | --- |
| How committed is this hospital to its patients and their loved ones? |  |  |  |  |  |
| How committed is this hospital to public health, or the health of the community around the hospital? |  |  |  |  |  |
| How likely are you to recommend this hospital to a loved one? |  |  |  |  |  |

**Table S1. Sample size estimates.**

| **Power** |  | **80%** |  |  | **85%** |  |  | **90%** |  |
| --- | --- | --- | --- | --- | --- | --- | --- | --- | --- |
| **Effect size** | **small** | **medium** | **large** | **small** | **medium** | **large** | **small** | **medium** | **large** |
| **Cohen's f-squared** | 0.01 | 0.06 | 0.14 | 0.01 | 0.06 | 0.14 | 0.01 | 0.06 | 0.14 |
| **Cohen's f** | 0.1 | 0.24 | 0.37 | 0.1 | 0.24 | 0.37 | 0.1 | 0.24 | 0.37 |
| **Cohen's d^1^** | 0.2 | 0.48 | 0.74 | 0.2 | 0.48 | 0.74 | 0.2 | 0.48 | 0.74 |
| **Odds ratio^2^** | 1.437 | 2.388 | 3.827 | 1.437 | 2.388 | 3.827 | 1.437 | 2.388 | 3.827 |
| **Sample size** | **n** | **n** | **n** | **n** | **n** | **n** | **n** | **n** | **n** |
| **Justification** | 966 | 163 | 72 | 1094 | 184 | 80 | 1265 | 211 | 90 |
| **Agency** | 789 | 135 | 61 | 901 | 153 | 68 | 1053 | 177 | 77 |
| **Family centered care** | 789 | 135 | 61 | 901 | 153 | 68 | 1053 | 177 | 77 |
| **Justification*Agency** | 966 | 163 | 72 | 1094 | 184 | 80 | 1265 | 211 | 90 |
| **Justification*Family centered care** | 966 | 163 | 72 | 1094 | 184 | 80 | 1265 | 211 | 90 |
| **Agency*Family centered care** | 789 | 135 | 61 | 901 | 153 | 68 | 1053 | 177 | 77 |

Sample size estimates calculated for main effects and interactions of policy elements using the n.multiway function from the R package easypower at significance level alpha = 0.05.

Effect Size Conversions:

**^1^**Cohen's *f to* Cohen's *d*:

$\boldsymbol{d}= 2 x \boldsymbol{f}$

**^2^**Cohen's *d to* Odds Ratio:

$\boldsymbol{log odds ratio}= \frac{\boldsymbol{d\pi}}{\sqrt{3}}$

$\boldsymbol{Odds Ratio}= exp(\frac{\boldsymbol{d\pi}}{\sqrt{3}})$

**^1^**Cohen, J. (1988). Statistical power analysis for the behavioral sciences (2nd ed.), Hillsdale, NJ: Erlbaum.

**^2^**Sánchez-Meca, J., Marín-Martínez, F., & Chacón-Moscoso, S. (2003). Effect-size indices for dichotomized outcomes in meta-analysis. Psychological Methods, 8(4), 448-467.

**Figure S1. Identification of unique inpatient facilities’ visitation policies.**


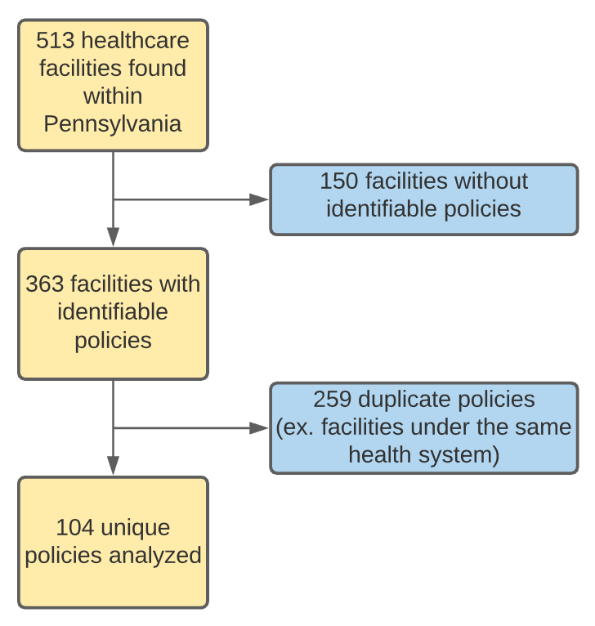


**Table S2. Multivariable models.**

| **Characteristic** | **OR (95% CI)** | **P value** |
| --- | --- | --- |
| **Justification** |  |  |
| Science vs. Authority | 1.30 (1.12, 1.51) | < 0.001 |
| Protection vs. Authority | 1.44 (1.24, 1.68) | < 0.001 |
| Protection vs. Science | 1.11 (0.949, 1.29) | 0.27 |
| **Agency** |  |  |
| Passive | 1.00 | 0.006 |
| Active | 1.16 (1.04, 1.29) |  |
| **Family centered care** |  |  |
| No | 1.00 | < 0.001 |
| Yes | 2.80 (2.51, 3.12) |  |
| **Urban or rural residence** |  |  |
| Urban | 1.00 | 0.045 |
| Suburban | 0.56 (0.35, 0.87) |  |
| Rural | 0.53 (0.31, 0.92) |  |
| Not reported | 0.15 (0.02, 1.27) |  |
| **Political party** |  |  |
| Democrat | 1.00 | 0.01 |
| Republican | 0.66 (0.41, 1.07) |  |
| Other | 0.42 (0.26, 0.69) |  |
| **At high risk for COVID-19** |  |  |
| No | 1.00 | 0.147 |
| Yes | 1.62 (0.99, 2.65) |  |
| Unsure, Not reported | 1.07 (0.62, 1.86) |  |
| **Revised Healthcare System Distrust Scale** |  |  |
|  | 0.87 (0.85, 0.90) | <0.001 |
